# Trajectories of care for children and adolescents with psychosocial problems: a 3‐year prospective cohort study

**DOI:** 10.1111/jcpp.13137

**Published:** 2019-10-20

**Authors:** Vera Verhage, Danielle E.M.C. Jansen, Josue Almansa, Charlotte Wunderink, Hans Grietens, Sijmen A. Reijneveld

**Affiliations:** ^1^ Department of Health Sciences University Medical Center Groningen University of Groningen Groningen The Netherlands; ^2^ Centre of Expertise Healthy Ageing Hanze University of Applied Sciences Groningen The Netherlands; ^3^ Department of Sociology and Interuniversity Center for Social Science Theory and Methodology (ICS) University of Groningen Groningen The Netherlands; ^4^ Centre of Expertise Healthy Ageing Friesland Mental Health Services Hanze University of Applied Sciences Groningen The Netherlands; ^5^ Department of Behavioral and Social Sciences University of Groningen Groningen The Netherlands

**Keywords:** Adolescence, mental health, service development, longitudinal studies

## Abstract

**Background:**

Care for children and adolescents with psychosocial problems is aimed at reducing problems. There may be a relationship between the intensity and duration of care provision and improvement of these outcomes, but evidence on this issue is lacking. We therefore examined the association between care trajectories based on duration and intensity of care for children, and the reduction in psychosocial problems after 3 years.

**Methods:**

We obtained a cohort of all children entering psychosocial care in one region (*n* = 1,378), the TAKECARE cohort, and followed it for 3 years, with five assessment rounds. Retention in the final round was 85.8%. Psychosocial problems were measured using the parent report of the Total Difficulty Score of the Strength and Difficulties Questionnaire (SDQ‐TDS). We constructed trajectories for intensity of care using growth mixture modelling and assessed the association between duration and intensity of care trajectories and SDQ‐TDS after 3 years.

**Results:**

After 3 months 60.6% of children and adolescents were receiving care, after 1 year 38.7% were receiving care and after 3 years 26.0%. Regarding intensity of care, three trajectories were found: one with minimal intensity during all 3 years, a second with initially medium intensity and strong reduction within 1 year, and a third with high intensity and a reduction after 1 year. Although the psychosocial problems of children and adolescents were reduced during the 3‐year period, the rate of decline was relatively less marked for children and adolescents with longer care trajectories.

**Conclusion:**

Overall, children and adolescents with psychosocial problems who received care had improved outcomes at follow‐up. However, increased provision of care does not automatically lead to reduction of problems, and although overall psychosocial problems are reduced, a substantial subgroup has longer lasting problems.

## Introduction

Up to one in five children worldwide experience psychosocial problems, that is, behavioral, emotional and social problems (Belfer, 2008; Kieling et al., 2011). Children with psychosocial problems experience poorer daily and social functioning (Rajmil et al., 2009) and have poorer physical health (Trzesniewski et al., 2006) and educational outcomes (Patel, Flisher, Hetrick, & McGorry, 2007; Uiters et al., 2014). Psychosocial problems in childhood are also related to health and developmental concerns in later life (Costello & Maughan, 2015; Patel et al., 2007), such as increased mental and physical health problems (Copeland, Wolke, Shanahan, & Costello, 2015), criminality (Fergusson, John Horwood, & Ridder, 2005), and social problems (Kessler, Walters, & Forthofer, 1998). Given the frequent occurrence of psychosocial problems among children and adolescents and the serious impact on their lives, it is important that they receive timely and effective treatment (Nanninga, 2018).

Randomized controlled trials on the effectiveness of psychosocial interventions have usually shown treatment in controlled settings for specific problems to have positive effects (e.g. Aldred, Green, & Adams, 2004; Lopata et al., 2010; Scott & O'Connor, 2012). However, the setting of such trials differs from the routine care provision in more naturalistic settings, where comorbidities are common and treatments typically less intensive (Thompson, 2009). Therefore, there is an urgent need for information on the characteristics of care provision in a naturalistic setting, which would make it possible to determine associations between psychosocial problems, the care delivered, and outcomes (Fein, 2017).

Studies on outcomes in naturalistic settings showed that children and adolescents with psychosocial problems who receive treatment experience some improvement, meaning less psychosocial problems were measured with SDQ at follow‐up (Deighton et al., 2016; Edbrooke‐Childs, Jacob, Law, Deighton, & Wolpert, 2015). In addition, another study showed a significant relationship between the number of treatment sessions (more than 8) and improvement in psychiatric symptoms (measured with the Child and Adolescent Psychiatric Assessment) at follow‐up (Angold, Costello, Burns, Erkanli, & Farmer, 2000). However, other studies reported different findings, including no association between treatment and improved outcomes at follow‐up (Jörg et al., 2012; Zwaanswijk, Verhaak, Van Der Ende, Bensing, & Verhulst, 2006), a very limited association (Trask & Garland, 2012), improvement limited to some subgroups, namely children with low to moderate externalizing problems (Tabone, Thompson, & Wiley, 2010), and even worse mental health outcomes after increased provision of treatment (Yampolskaya, Sharrock, Clark, & Hanson, 2017). A reason for these heterogeneous findings may include a rather crude assessment of treatment, that is, only dichotomized as yes/no treatment (Tabone et al., 2010; Trask & Garland, 2012; Zwaanswijk et al., 2006). Hence, there is a clear need for well‐designed studies with detailed descriptions of the characteristics of the care provided in order to provide insight into the long‐term outcomes of care for children and adolescents.

Therefore, we aimed to describe the duration and intensity of care trajectories and assess longer‐term outcomes, that is, problem reduction after 3 years, by duration and intensity of the care trajectory. We did so by using a longitudinal design on a cohort that included the entire spectrum of care for children and adolescents with psychosocial problems and detailed descriptions of the duration and intensity of the care provided.

## Methods

### Cohorts

This study formed part of the TAKECARE study. TAKECARE is a prospective cohort study conducted in the northern Netherlands to investigate the care offered to all children and adolescents with psychosocial problems entering care in a delineated area (*n* = 1,378). In the Netherlands, children, adolescents and their families can receive psychosocial care from professionals within various types of services, including Preventive Child Healthcare (PCH), Child and Adolescent Social Care (CASC), and Child and Adolescent Mental Healthcare (CAMH). In PCH, doctors and nurses provide light psychosocial or parental support (ambulatory/outpatient or home‐based) to children and families with mild problems. In case of more severe problems, PCH professionals refer children and families to specialized care (i.e. CASC or CAMH); other health‐care professionals such as general practitioners may refer too. In CASC, child (social) workers and child development specialists provide specialized care (i.e. trauma support, supportive independent living, foster care) to children and families. In CAMH, psychologists and psychiatrists provide children and families with specialized care for psychosocial problems and psychiatric disorders (Nanninga, 2018).

The cohort consisted of children and adolescents who were either entering care—PCH, CASC or CAMH—for psychosocial problems (care cohort), or were part of the general population in the same region (community cohort). Inclusion criteria for the care and community cohort were: aged between 4 and 18 years old, estimated IQ higher than 70, the parents and/or the child being reasonably able to understand Dutch and residing in one of the three northern Dutch provinces. Regarding the care cohort, receiving psychosocial care from one (or more) of the four participating care organizations (excluding routine pediatric monitoring) was a prerequisite for eligibility.

Participants in the community cohort were randomly selected from primary, secondary and intermediate vocational education schools (*n* = 666). Participants in the care cohort were recruited from four care organizations, focusing respectively on PCH (*n* = 1), CASC (*n* = 1) and CAMH (*n* = 2), in order to obtain a representative cohort of care profiles in terms of problem severity, duration and type of care (*n* = 1,382).

Parents and professionals received the first questionnaire (T1) directly after inclusion. Baseline measurements were taken from May 2011 until April 2013. The questionnaires of the second (T2), third (T3), fourth (T4) and fifth (T5) rounds were sent 3, 12, 24 and 36 months after the first questionnaire, respectively. Involved professionals received questionnaires for as long as the participant received psychosocial care from one of the four collaborating organizations. Detailed information on the procedures and measures used in the questionnaires for the parent, adolescent or professional has been given earlier (Verhage, Noordik, Knorth, & Reijneveld, 2016).

From the catchment area, 2,615 participants were potentially eligible. Of the potentially eligible participants, 2,082 could be approached, of whom 174 were excluded after telephone contact with research assistants, due to their not meeting the inclusion criteria. Of the remaining 1,908, 1,382 (56.6% of those eligible) were included in the study (Verhage et al., 2016). With regard to non‐participants, we collected data on some baseline characteristics and we used a single question about the severity of emotional and behavioral difficulties.

Of the 1,382 baseline participants, 1,283 parents and/or adolescents (92.8%) participated in the second round, 1,256 (90.8%) in the third round, 1,202 (86.9%) in the fourth round, and 1,177 (85.2%) in the final round. At baseline, differences between respondents and non‐respondents in age, gender, rural/urban area and experienced impairment (measured by the impact scale of the Strength and Difficulties Questionnaire (SDQ)) (Van Widenfelt, Goedhart, Treffers, & Goodman, 2003) were small or trivial. We could not test for differences in total SDQ scores since we only asked the non‐responders a limited set of questions, in order to restrict the required time investment.

Attrition at follow‐up was somewhat higher for children with a non‐Dutch ethnicity and for children who lived in a low‐income household or without both biological parents. However, there was no difference between respondents and non‐respondents in terms of psychosocial problems (Verhage et al., 2016). Written and informed consent was given by those who actually participated in the study. The Medical Ethical Committee of the University Medical Center Groningen assessed the design of the study and gave approval without requiring full assessment.

### Procedure and measures

All potentially eligible participants received oral and written information about the study via the four care organizations. Research assistants then invited parents and children to participate in the study by telephone. The questionnaires were sent by e‐mail or by surface mail, according to the participants’ preference, with a reminder after 1 and 2 weeks. To prevent attrition, parents and children were frequently reminded to complete the questionnaire, and after every completed questionnaire they were rewarded with a gift token. The professionals involved received questionnaires for as long as the subjects received psychosocial care from one of the four collaborating organizations.

### Outcome

The outcome concerned the difference between the SDQ Total Difficulty Score (TDS) of the child/adolescent at the start of the care trajectory (T1) and 3 years thereafter (T5), reported by the parent/caregiver. The SDQ is a brief questionnaire for the identification of psychosocial problems. The psychometric properties of the SDQ have been shown to be good in different settings and in a number of countries, including the Netherlands (Crone, Vogels, Hoekstra, Treffers, & Reijneveld, 2009; Goodman, Ford, Simmons, Gatward, & Meltzer, 2000). The SDQ‐TDS consists of 25 items that describe positive and negative attributes of children with regard to emotional problems, behavioral problems, hyperactivity, and peer problems (Cronbach's α = .84). We excluded four respondents with missing data on the SDQ‐TDS at baseline, resulting in *n* = 1,378 respondents for the analyses.

### Predictors

Care trajectories were defined based on the duration and intensity of the psychosocial care received in a 3‐year period; this could involve one or multiple intervention(s) (with a maximum of five different interventions per measurement round). As the first measurement round (T1) concerned the start of the care trajectory, there was no score for duration or intensity at T1.

*Duration of care* was operationalized in two ways. First, by counting the number of children and adolescents that received care or who received again care at the time of the different measurement rounds (yes/no). Second, by looking at the total time period during which the care was provided. Subsequently, duration of care regards the total time period (in months) between the first day of care and the last day of care, within the 3‐year follow‐up period. The total duration of care was classified as (a) ‘0–3 months,’ (b) ‘3 months to 1 year,’ and (c) ‘1 year or longer.’

*Intensity of care* regarded the amount of care, in number of hours, received during the full period between the current measurement and the preceding one. It included all kinds of treatment offered by one of the four collaborating organizations, for example parental coaching, trauma support or psychotherapy. It was based on monthly reports from all professionals providing care. We could not use administrative data because the recorded data differed strongly between types of care providers, restricting the potential for cross‐sectoral analyses. Missing values regarding the amount of care (if receiving care) were deduced from the mean intensity of the treatments reported for that round (12.5%). In cases where professionals mentioned that they offered care but the number of hours was missing, values were deduced from the mean intensity for the other reported respondents for that round.

### Potential confounders

Potential confounders regarded age (Reijneveld, Brugman, Verhulst, & Verloove‐Vanhorick, 2004; Simpson, Cohen, Bloom, & Blumberg, 2009), gender (Bussing, Zima, Gary, & Garvan, 2003; Cuffe, Moore, & McKeown, 2009), family composition (Costello, He, Sampson, Kessler, & Merikangas, 2014), educational level (Reijneveld et al., 2004), social support (Nanninga, Jansen, Knorth, & Reijneveld, 2015), parenting skills (Nanninga et al., 2015), and problem severity measured with the SDQ‐TDS (Bussing et al., 2003; Klein Velderman, Crone, Wiefferink, & Reijneveld, 2009), all measured during the first measurement round (T1: entry into care). These potential confounders were chosen based on the indicated prior empirical research.

#### Age

Age at baseline was categorized as 4–11 and 12–18, as in the Dutch educational system primary school age approximately includes ages 4–11 and secondary school age includes ages 12 and over.

#### Family composition

Family composition was measured by asking the parent with whom the child lived. This was categorized into ‘biological two‐parent family’ and ‘other’ (e.g. living with one parent, a blended (step) family or a foster family).

#### Educational level of the mother/caregiver

Educational level of the mother/caregiver was based on the highest educational level achieved by the mother/caregiver (for example when a child lived with a foster family).

#### Social support

Social support was measured by a subscale of the Family Questionnaire (FQ) (Van der Ploeg & Scholte, 2008). The subscale measures support from relatives, friends and neighbors, with nine items on a five‐point Likert scale.

#### Parenting skills

Parenting skills were measured by two subscales of the nine‐item version of the Alabama Parenting Questionnaire (APQ) (Elgar, Waschbusch, Dadds, & Sigvaldason, 2007). Research shows that of the three original subscales, inconsistent discipline and poor supervision are associated with care enrolment and that there is no association with positive parenting (Nanninga et al., 2015). The subscales were measured on a five‐point Likert scale.

### Analyses

First, we described the background characteristics of the children and adolescents in the cohort. Second, we described the care provided in terms of duration and intensity based on the T2, T3, T4 and T5 measurement rounds. For the latter, we used Growth Mixture Models (GMM) with random intercepts. Time was modelled as a categorical variable. Thus, each trajectory class is defined by the intensity mean per round plus a random intercept. Random intercept variances and residual variances were estimated differently per class. Also, residual variances within each class were allowed to vary per round. To set the optimal number of classes, the Bayesian information criterion (BIC) was calculated; the model with the lowest BIC was used. Third, we assessed the association between the total duration of care and various intensity trajectories (class‐trajectory membership) of care, and problem reduction after 3 years. We performed a multivariate regression analysis, with the SDQ‐TDS at 3 years as the dependent variable, and adjusted for baseline SDQ‐TDS *(*i.e. the crude model). Next, we adjusted for baseline SDQ‐TDS and for all potential confounders based on theory, with the enter‐method (i.e. adjusted model).

## Results

### Background characteristics of the cohort

At baseline 60.1% of the cohort was aged between 4 and 11 years old. The average age was 10.9 years and 46.7% were female. The majority of the children and adolescents did not live with both biological parents (52.6%). The mean SDQ‐TDS at baseline as reported by the parent/caregiver was 15.7 and differed by duration of care; with a mean SDQ‐TDS of 13.1 for children and adolescents with a care duration between 0 and 3 months, 15.5 when care duration was between 3 and 12 months, and 17.0 for a care duration longer than 12 months (*p* < .001). In Figure S1 in the Supporting Information, the mean SDQ‐TDS is shown per subgroup of care duration during the 3‐year period. Children and adolescents with a care duration shorter than 3 months had a mean SDQ‐TDS of 13.1 at T1 and 7.9 at T5 (Table 1). For a care duration between 3 and 12 months the mean SDQ‐TDS was 15.5 at T1 and 10.6 at T5, while for a care duration of more than 12 months the mean SDQ‐TDS was 17 at T1 and 13.9 at T5 (see Figure S1).

**Table 1:** Background characteristics of the cohort at baseline

	T1 Entry into care (*n* = 1,378)^a^	
	*N*	*%*
Age		
4–11 years old	828	60.1
12–18 years old	550	39.9
Gender		
Girls	644	46.7
Boys	734	53.3
Family composition		
Living with both biological parents	652	47.4
Other	723	52.6
Educational level of the mother/caregiver		
Primary education	70	5.4
Lower levels of secondary education	275	21.2
Higher levels of secondary education	706	54.4
Senior vocational education	186	14.3
University	61	4.7

	Mean	*SD*
Problem severity (SDQ‐TDS) (range 0–40; 0 = no problems)	15.7	6.6
Problem severity (SDQ‐TDS) by duration		
Duration 0–3 months (*n* = 347)	13.1	6.3
Duration 3–12 months (*n* = 264)	15.5	6.3
Duration > 12 months (*n* = 564)	17.0	6.4
Parenting skills (APQ)		
Poor supervision (range 1–5; 1 = poor supervision)	1.9	0.8
Inconsistent disciplining (range 1–5; 1 = inconsistent disciplining)	2.3	0.7
Social support (range 1–45; 1 = low social support)	36.8	7.6

Numbers do not always add up to *N* = 1,378 due to missing data (family composition: *n* = 3; educational level of the mother/caregiver: *n* = 80).

### Duration and intensity of care

**Figure 1:**
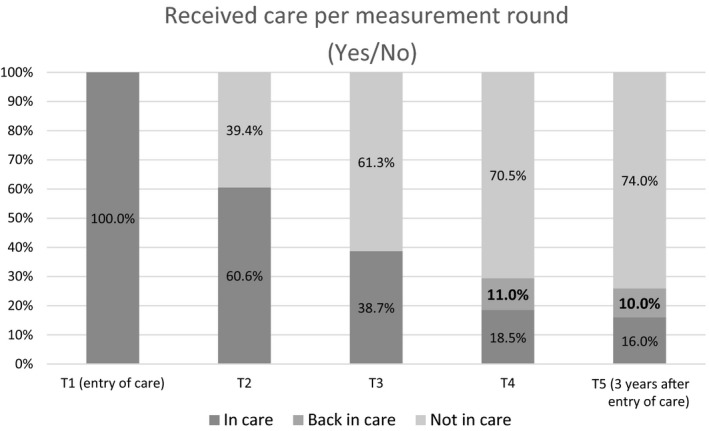
Number of children and adolescents that received care in the various measurement rounds. The bold numbers at T4 and T5 denote the children and adolescents who did not receive care in the previous measurement round; for example, in measurement round 5 (T5), 26% of the children and adolescents received care, of whom 10% did not receive care at T4

The care provided was defined based on the *duration* during a 3‐year period and on the *intensity* of the contact with the professional. In Figure 1 the children and adolescents who received care or who received care again are shown per measurement round. At entry into care (T1), 1,378 (100%) children and adolescents received care. After 3 months (T2) this had decreased to 60.6%, after 1 year (T3) to 38.7%, after 2 years (T4) to 29.5% and after 3 years (T5) to 26.0%.

Regarding *intensity* of care, the three‐class model of trajectories was selected based on model fit (Table 2). This option had the lowest values of AIC and BIC; moreover, the four‐class solution did not converge properly.

**Table 2:** Trajectories of intensity of care: number of classes of intensity as identified and fit statistics

# Classes	Class size (%) per class			BIC	AIC	Entropy
	1	2	3			
1	100			35,368.2	35,321.4	
2	57.4	42.6		24,488.1	24,404.8	0.961
3	44.2	38.1	17.7	22,201.3	22,066.0	0.922

BIC, Bayesian information criterion; AIC, Akaike information criterion.

**Figure 2:**
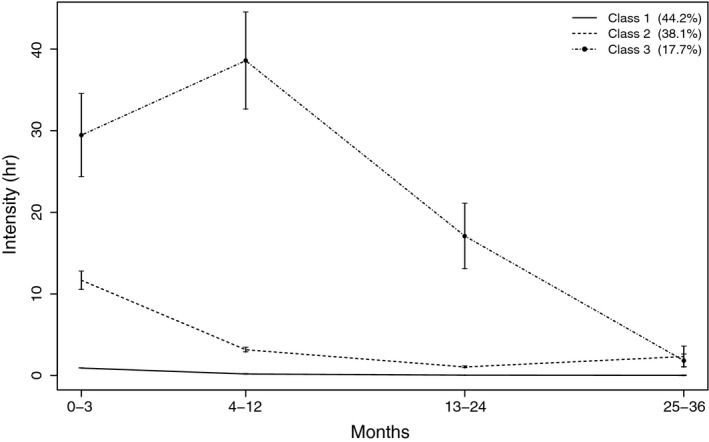
Trajectories of intensity of care in hours per month, regarding the period before the assessment. The intervals at 3–36 months concern 95% confidence intervals of the estimated means within trajectories (residual variances, within‐person variability; confidence intervals for class 1 were very narrow)

Figure 2 shows the results for the three‐class model. Class 1 (44.2%) consistently had a minimal intensity of care during all 3 years, with very low variability. Class 2 (38.1%) started with a medium intensity of care and saw the strongest reduction within 1 year. Class 3 (17.7%) started with a high intensity of care and saw a reduction in intensity after 1 year.

### Outcomes after 3 years predicted by duration and intensity

In general, we found a reduction in problem severity at baseline (mean SDQ‐TDS 15.7) and 3 years later (mean SDQ‐TDS 11.7*)*. Figure S2 shows the mean SDQ‐TDS scores per measurement round during the 3‐year period for the care cohort and also for the community (reference) cohort. Regression analyses (Table 3) showed that children who received care for a period between 3 and 12 months or longer than 12 months had higher levels of psychosocial problems after 3 years than children with a shorter duration of care (0–3 months). In other words: the problem reduction was less marked for children and adolescents with longer care trajectories. When adjusting for the confounders (background characteristics, social support and parenting skills), the problem reduction 3 years after the start of the care trajectory was still smaller for longer trajectories (Table 3). No such association was found for intensity of care.

**Table 3:** Associations of duration and intensity classes and SDQ‐TDS after three years. Results of regression analyses with SDQ‐TDS at T5 as outcome adjusted for SDQ‐TDS at T1, and for the covariates as shown

	Crude			Adjusted^a^		
	*B*	95% CI	*p*	*B*	95% CI	*p*
Duration (reference: 0–3 months)						
3–12 months	1.263	(0.330; 2.196)	.008	1.582	(0.648; 2.516)	.001
Longer than 12 months	3.782	(2.978; 4.585)	<.001	4.011	(3.208; 4.813)	<.001
Intensity (reference: Class 1)						
Class 2	0.458	(−0.264; 1.180)	.214	0.467	(−0.254; 1.189)	.204
Class 3	0.649	(−0.298; 1.596)	.179	0.656	(−0.296; 1.608)	.177
Baseline SDQ‐TDS^b^	0.537	(0.486; 0.587)	<.001	0.504	(0.451; 0.558)	<.001
Age (at baseline)				−1.247	(−1.937; −0.557)	<.001
Gender (girls vs. boys)				−0.097	(−0.749; 0.555)	.771
Family composition (both biological parents vs. other)				−0.095	(−0.745; 0.554)	.774
Educational level mother (other vs. low)				−0.140	(−0.486; 0.206)	.427
Social support (vs. low social support)				−0.048	(−0.093; −0.002)	.040
Inconsistent discipline (vs. consistent)				0.636	(0.179; 1.094)	.006
Poor supervision (vs. good)				−0.061	(−0.545; 0.423)	.806

a Adjusted for children's age, gender, family composition, education level of the mother, social support, parenting skills and baseline SDQ. Crude was only adjusted for baseline SDQ.

b SDQ at T1: mean 15.7.

## Discussion

We examined the duration and intensity of care trajectories for children and adolescents who received psychosocial care and assessed the outcomes for each trajectory after 3 years. We found that many children received care shorter than 3 months, but after 3 years about a quarter still received care or received again care. Regarding intensity of care, children and adolescents were classified into three trajectories, one with minimal intensity during all 3 years, a second with initially medium intensity and strong reduction within 1 year and a third one with high intensity and a reduction after 1 year. Overall, the psychosocial problems of children and adolescents were reduced during the 3‐year period. In comparison with the community cohort, children and adolescents who received care had a higher problem level at the start (care cohort: mean SDQ‐TDS 15.7; community cohort: mean SDQ‐TDS 7.2), and after receiving care still had a higher problem level (mean SDQ‐TDS 11.7) than the community cohort (mean SDQ‐TDS 6.2). Finally, the reduction in the psychosocial problem levels of children and adolescents with longer care trajectories was relatively smaller.

Our finding that care was frequently provided for <3 months confirms that most interventions for children and adolescents with psychosocial problems focus on short‐term treatment gains (Mulqueen, Bartley, & Bloch, 2015; Wehry, Beesdo‐Baum, Hennelly, Connoly, & Strawn, 2015). Moreover, the SDQ scores of children and adolescents with a short duration of care further improve after care has ended after 3 months. This finding indicates that care professionals can to a considerable degree provide a good estimate of the prognosis of children and adolescents who receive psychosocial care, and it suggests a lasting effect of short care (Birmaher et al., 2000). Research on the specific skills that help to reach such estimates of prognosis is scarce, and this evidently requires further study. The same is true regarding the components of care that contribute to the sustainability of the effects of care. Research on the distinct techniques delivered by the professional (practice elements) and aspects of the intervention design and service delivery system (program elements) (see e.g. Visscher et al., 2018) could be promising in addressing these issues.

About a quarter of children and adolescents still received care after 3 years and SDQ scores of children and adolescents with long trajectories did not improve after 24 months. A likely underlying cause is treatment resistance: problems do not resolve, and therefore either the client or the professional want to continue treatment, or both of them do. Research from Tabone et al. (2010) showed a significant relationship between mental health services and beneficial outcomes, but only for children/adolescents with mild or moderate problems. A total resolution of problems may therefore simply not be achievable for a substantial group of children and adolescents. The alternative explanation – longer types of care being less effective – seems less likely, although it cannot be excluded and requires further study. Our study adds that trajectories of care may differ considerably, also within the group receiving longer term care.

Regarding the intensity trajectories, we found that the intensity of care for two out of the three trajectories was particularly high in the first period. These findings corroborate earlier findings showing that the number of services provided was high at the start of care and then decreased (Yampolskaya et al., 2017), and can be interpreted as proving that for a majority of clients, professionals start with rather intensive care. Our finding of a third, persistently low‐intensity trajectory is new and calls for further research on its reasons. An explanation could be that, already early in the care trajectory, professionals are able to estimate that the problems are chronic in nature and then decide to provide mostly supportive care. A further assessment of the contents of the care as provided may prove useful here.

We did not find any association between intensity of care and problem reduction after 3 years, which is in line with two earlier studies (Andrade, Lambert, & Bickman, 2000; Zwaanswijk et al., 2006). A possible explanation is that children and adolescents may not have received all the care they needed, resulting in continued care without improvement. Another explanation may be that care addressed problems that were not fully captured by the SDQ, such as intra‐parental conflict, that was a risk for psychosocial health but did not actually impact SDQ scores. However, this does not explain why an association with duration has been found. And third, some problems may simply be treatment resistant, as discussed before.

Regarding outcomes, we found that the psychosocial problems of the children and adolescents reduced over time. The SDQ scores of children and adolescents who receive care remain higher than the scores of the community cohort, but it is promising that their mean SDQ scores decrease from the borderline to the normal range. This overall trend of problem reduction is positive and might be interpreted as an indicator of the quality of care, although the problem reduction varies between subgroups, and regression to the mean may also contribute (Barnett, van der Pols, & Dobson, 2005). Ideally, this long‐term trend should be confirmed in experimental studies, but this is evidently very difficult to realize. Overall, our findings suggest a properly functioning triage in the care system: first children and adolescents with longer trajectories experienced a lower rate of problem reduction and second after short treatment, problem reduction continued at follow‐up. These findings correspond with earlier research by Nanninga, Jansen, Knorth, and Reijneveld (2018), who found that the system of psychosocial care functions as intended regarding the distribution of type and severity of problems across care types (Nanninga et al., 2018). Overall, increased provision of care does thus not automatically lead to reduction of problems, and although overall psychosocial problems are reduced, a substantial subgroup has longer lasting problems.

### Strengths and limitations

This study has considerable strengths. We had a large cohort that represents all of the psychosocial care provided to children and adolescents, with a long follow‐up (3 years) and high retention, and extensive measurements allowing us to control a wide range of covariates. Furthermore, detailed measures of duration and intensity were used to define the care trajectories. Our study also has some limitations. First, the response rate at inclusion was relatively low. However, differences by response status were small, limiting the likelihood of selection bias (Verhage et al., 2016). Second, we assessed the outcomes of the services provided in an observational design, which limited the potential for inferences on the effectiveness of care. Third, we used the SDQ‐TDS as an outcome measure, so we probably captured only a part of the problems leading to a care trajectory, a more in‐depth diagnostic approach was, however, not possible, considering the broad spectrum of services that is included in this study.

### Implications

With this study we unraveled a little piece of the puzzle of associations between psychosocial problems, the care provided, and the outcomes. We found that the psychosocial problems of children and adolescents that received care were generally reduced over time, which is promising. However, the workload of professionals is often mainly determined by the care for children with severe and lasting problems, making the professionals relatively pessimistic about the prognosis of children (Costello & Maughan, 2015). Our findings could therefore help achieve a more balanced view of prognosis.

Furthermore, we found a rather large variation in the duration and intensity patterns of care, which calls for further research on the determinants of this variation and their impact on outcomes. Future research could therefore focus on the factors that influence the preference for short versus long, or intensive versus less intensive interventions. The persistence of psychosocial problems while being in treatment requires further study, in particular as to whether better aligning of treatment elements with problems could lead to better outcomes, or whether some problems are chronic in nature in any case, as several authors have suggested (Anderson & Lowen, 2010; Okado, Ewing, Rowley, & Jones, 2017). Moreover, detailed information about the components of treatments may help to improve their effectiveness (Evenboer, Huyghen, Tuinstra, Knorth, & Reijneveld, 2016). Future research could also be based on administrative data instead of on professional reports. This requires standardized recording of these data, to allow for cross‐sectional analyses.

Finally, future research should include other outcome measures as well, because care for children and adolescents is not always solely aimed at problem reduction (Jacob et al., 2017). Examples include outcomes that focus on individual outcomes (Costello & Maughan, 2015) or on societal participation.

**Key points**

- Up to one in five children worldwide experience psychosocial problems, i.e. behavioral, emotional and social problems.
- Evidence on the outcomes of psychosocial care obtained in naturalistic settings is scarce and inconclusive.
- We found that the psychosocial problems of children and adolescents who received care were reduced during the 3‐year period, but relatively less so for children and adolescents with longer care trajectories.
- Our findings underscore the importance of examination of the outcomes of psychosocial care for the children and adolescents with the most severe and lasting problems.

## Supporting information


**Figure S1.** Trajectories of SDQ‐TDS for the care cohort (*n* = 1,283) by duration of the received care.
**Figure S2.** Trajectories of SDQ TDS for the care (*n* = 1,283) and the community cohort (*n* = 666).
