# Supplementary material for: Trajectories of care for children and adolescents with psychosocial problems: a 3‐year prospective cohort study
Source: J Child Psychol Psychiatry. 2019 Oct 20;61(5):556–64. doi: 10.1111/jcpp.13137 (PMC7216877; doi:10.1111/jcpp.13137)
Supplement: Supplementary file 1 — Figure S1. Trajectories of SDQ‐TDS for the care cohort (n = 1,283) by duration of the received care. Figure S2. Trajectories of SDQ TDS for the care (n = 1,283) and the community cohort (n = 666). [file JCPP-61-556-s001.docx]

**Supporting information – Trajectories of care for children and adolescents with psychosocial problems: a three-year prospective cohort study – by Verhage *et al.***

**Figure S1.** Trajectories of SDQ-TDS for the care cohort (n=1,283) by duration of the received care

Footnote: SDQ cutoff points are: normal (0-13), borderline (>13-16) and, abnormal (>16).

**Figure S2.** Trajectories of SDQ TDS for the care (n=1,283) and the community cohort (n=666)

Footnote: SDQ cutoff points are: normal (0-13), borderline (>13-16), and abnormal (>16).
